# GLP-1 based therapeutics: simultaneously combating T2DM and obesity

**DOI:** 10.3389/fnins.2015.00092

**Published:** 2015-03-20

**Authors:** Kristy M. Heppner, Diego Perez-Tilve

**Affiliations:** ^1^Division of Diabetes, Obesity and Metabolism, Oregon National Primate Research Center, Oregon Health and Science UniversityBeaverton, OR, USA; ^2^Department of Medicine, Metabolic Diseases Institute, University of CincinnatiCincinnati, OH, USA

**Keywords:** GLP-1, obesity, diabetes, CNS, insulin

## Abstract

Glucagon-like peptide-1 (GLP-1) enhances meal-related insulin secretion, which lowers blood glucose excursions. In addition to its incretin action, GLP-1 acts on the GLP-1 receptor (GLP-1R) in the brain to suppress feeding. These combined actions of GLP-1R signaling cause improvements in glycemic control as well as weight loss in type II diabetes (T2DM) patients treated with GLP-1R agonists. This is a superior advantage of GLP-1R pharmaceuticals as many other drugs used to treat T2DM are weight neutral or actual cause weight gain. This review summarizes GLP-1R action on energy and glucose metabolism, the effectiveness of current GLP-1R agonists on weight loss in T2DM patients, as well as GLP-1R combination therapies.

## Introduction

Obesity is a global health problem and increases the risk of developing type II diabetes mellitus (T2DM), cardiovascular disease, and dyslipidemia. These metabolic complications caused by obesity result in a decreased lifespan (Fontaine et al., 2003; Olshansky et al., 2005). The cost of treating obesity-related diseases has reached epic proportions and is estimated to cost $147 billion in the US alone (Finkelstein et al., 2009). Many treatments approved for T2DM treatment such as sulphonylureas and thiazolidinediones (TZDs) improve glucose metabolism but have the unwanted side effect of causing weight gain (Thule and Umpierrez, 2014) and (Kahn et al., 2006; Home et al., 2009; Ryan et al., 2011). As the majority of patients with T2DM are also obese, medications that exacerbate weight gain are not ideal. Moreover, small reductions in body weight alone can have beneficial effects on blood pressure as well as glucose and cholesterol metabolism (Klein et al., 2004; Wing et al., 2011). Therefore, therapies that promote weight loss and simultaneously improve glucose metabolism are superior treatments for obese patients with T2DM.

Glucagon-like peptide-1 (GLP-1) is a gastrointestinal hormone as well as a neurotransmitter. GLP-1 was initially discovered for its ability to enhance glucose-stimulated insulin secretion (GSIS) (Kreymann et al., 1987; Mojsov et al., 1987). Shortly after this, it was demonstrated that GLP-1 suppresses food intake through CNS-mediated mechanisms (Turton et al., 1996; Tang-Christensen et al., 1998). Consistent findings have shown that GLP-1R agonism promotes weight loss and improves glucose homeostasis in rodents, monkeys and humans (reviewed in Barrera et al., 2011a). This dual action has made GLP-1 analogs superior therapeutics for the treatment of T2DM.

## The anatomical distribution of the endogenous GLP-1 system

GLP-1 is derived from the preproglucagon gene. In the periphery, posttranslational processing of preproglucagon in the alpha-cells of the pancreas produces glucagon and a small amount of GLP-1 whereas the main source of peripheral GLP-1 production occurs through the posttranslational processing of preproglucagon in the L-cells of the gut (reviewed in Holst, 2007 and Campbell and Drucker, 2013). The GLP-1R is located in a number of peripheral tissues including the pancreas, gastrointestinal tract, kidney, lung and heart (Thorens, 1992; Bullock et al., 1996; Pyke et al., 2014). The GLP-1R is a class B1 G-protein coupled receptor that predominately couples to a Gα_*s*_ subunit leading to the activation of adenylyl cyclase (Kirkpatrick et al., 2012; Willard and Sloop, 2012).

In the brain, preproglucagon expressing neurons are localized to the brainstem in regions including the nucleus of the solitary tract (NTS) and ventrolateral medulla (Merchenthaler et al., 1999) and these neurons produce a number of peptides including GLP-1, oxyntomodulin, and glucagon-like peptide-2 (Holst, 2007). CNS preproglucagon distribution is similar in rodents, monkeys, and humans (Merchenthaler et al., 1999; Vrang and Grove, 2011; Zheng et al., 2015). Brainstem preproglucagon expressing fibers project to many areas of the brain with the most abundant projections to areas hypothalamic areas that control energy homeostasis including the arcuate nucleus (ARC), paraventricular nucleus (PVN), and dorsomedial nucleus (DMH) (Jin et al., 1988). The distribution of the GLP-1R is similar to preproglucagon fiber projections with high expression in hypothalamic areas that regulate energy homeostasis, both in rodents and non-human primates (Merchenthaler et al., 1999; Heppner et al., 2015).

## GLP-1R-mediated action on glucose metabolism: peripheral vs. CNS actions

GLP-1R signaling plays a critical role in the maintenance of glucose homeostasis as deletion of the GLP-1R in mice results in impaired glucose tolerance (Scrocchi et al., 1996) and physiological levels of GLP-1 enhance meal-related insulin secretion in humans (Kreymann et al., 1987; Mojsov et al., 1987). Although it is well established that the endogenous GLP-1R system is essential for glycemic control, the specific GLP-1R population that mediates this effect is still a matter of debate. In the periphery, GLP-1 acts directly on pancreatic islets to enhance GSIS (Mojsov et al., 1987) and inhibit glucagon release (Komatsu et al., 1989). A study in transgenic animals demonstrates that selectively restoring pancreatic GLP-1Rs in GLP-1R null mice normalizes glucose tolerance in these animals suggesting that GLP-1R expression in the pancreas is sufficient to maintain normal glucose metabolism (Lamont et al., 2012). In contrast, another study demonstrated that transgenic mice with a beta-cell specific deletion of the GLP-1R have impaired intraperitoneal (ip) glucose tolerance but maintain normal oral glucose tolerance (Smith et al., 2014). These data demonstrate that GLP-1R signaling in the beta cells reduces hyperglycemia but other GLP-1Rs, possibly on neural tissue, are involved in mediating the incretin action of GLP-1.

In addition to this direct control of beta cells, GLP-1R signaling plays a role in the control of glucose homeostasis by regulating the activity of neurons both in the peripheral and central nervous system. Thus, blockade of hepatic portal GLP-1R signaling causes glucose intolerance in rats, suggesting that GLP-1Rs located on nerve terminals in the hepatic portal vein contribute to the incretin action of GLP-1 (Vahl et al., 2007). Furthermore, GLP-1 stimulates preganglionic vagal neurons projecting to the pancreas (Wan et al., 2007), raising the possibility that this mechanism may contribute to GLP-1 stimulation of insulin secretion.

Rodent studies involving pharmacological manipulation of GLP-1R in the brain by intracerebroventricular (ICV) administration of agonists and antagonists demonstrate a role for brain GLP-1R signaling in the control of glucose metabolism. However, this regulation appears to be complex, likely as a result of the contribution of multiple sites throughout the CNS that target distinct elements in peripheral tissues that play a role in the control of glucose homeostasis. For instance, central administration of GLP-1R agonists increases the ability of insulin to suppress endogenous glucose production (Knauf et al., 2005; Sandoval et al., 2008; Burmeister et al., 2012). Interestingly, this occurs despite a reduction in glucose uptake in muscle (Knauf et al., 2005), which is consistent with the increased muscle glucose uptake exhibited by mice lacking GLP-1R expression (Ayala et al., 2009). The complexity of the contribution of CNS-GLP-1R to the control of glucose homeostasis is accentuated by marked interspecies differences. For example, chronic ICV infusion of the GLP-1R antagonist exendin-9 impairs glucose tolerance in rats, supporting a role for CNS GLP-1R signaling in the maintenance of glycemic control (Sandoval et al., 2008; Barrera et al., 2011b). In contrast, another study in rats demonstrated that acute injection of the GLP-1R agonist exendin-4 (Ex-4) increases baseline glucose levels in rats which is due to the activation of the sympathetic nervous system (Perez-Tilve et al., 2010). These data indicate that GLP-1R signaling regulates the activity of different neuronal populations involved in the control of specific aspects contributing to glucose homeostasis. However, recent evidence demonstrates that this regulation may not be necessary for the maintenance of whole body glucose metabolism, at least in mice. Hence, mice lacking GLP-1R only in the peripheral nervous system (PNS) or central nervous system have normal ip and oral glucose tolerance as compared to controls (Sisley et al., 2014). Furthermore, chronic treatment with the GLP-1 mimetic liraglutide induces similar improvements on glucose tolerance despite the loss of PNS or CNS-GLP-1R expression, indicating that non-neuronal GLP-1R signaling is sufficient for the long-term improvements in glucose homeostasis induced by liraglutide in mice (Sisley et al., 2014).

## The endogenous GLP-1 system in the regulation of energy homeostasis

The endogenous GLP-1 system is densely populated in areas that control energy homeostasis placing it in the neuroanatomical position to regulate food intake and body weight. GLP-1 producing neurons in the brainstem are activated following a large satiating meal suggesting that these neurons relay satiety signals to higher brain regions (Kreisler et al., 2014). Knockdown of preproglucagon expression in the hindbrain as well as central GLP-1R antagonism results in hyperphagia and body weight gain in rats indicating that these neurons are essential for maintenance of normal body weight (Barrera et al., 2011b). Global knockout of the GLP-1R produces no body weight or food intake phenotype in chow-fed mice (Scrocchi et al., 1996). However, when these mice are placed on a high-fat diet (HFD) the *Glp1r−/−* animals are leaner than wild-type (WT) controls (Hansotia et al., 2007; Wilson-Perez et al., 2013), a phenotype that goes against the role of GLP-1R signaling in the control of body weight. However, the GLP-1R is also involved in adipogenesis in the periphery, which may account for this discrepancy (Challa et al., 2012). To further dissect the role of the endogenous CNS GLP-1R system, conditional CNS specific knockout models were created. Similar to global *Glp1r−/−* mice, animals with deletion of the GLP-1R in vagal afferent/efferent nerves, *Phox2b-Cre Glp1r^flox/flox^*, as well as animals with a deletion of the GLP-1R in the CNS, nestin-*Cre Glp1r^flox/flox^*, maintain a similar body weight and food intake as control mice on both chow and HFD (Sisley et al., 2014). These findings challenge whether endogenous PNS or CNS GLP-1R signaling is essential for maintaining normal energy homeostasis. Again, these discrepancies in the literature investigating the role of GLP-1R signaling in the control of energy homeostasis could be partially due to mammalian species differences, mice vs. rats, or differences in methodologies used to manipulate GLP-1R signaling.

## Pharmacological effects of GLP-1R agonism on energy homeostasis

Despite controversial findings over the role of the endogenous GLP-1 system in maintaining energy homeostasis, consistent findings demonstrate that pharmacological administration of GLP-1R agonists suppresses body weight across a number of species. The specific sites of action and underlying molecular mechanisms that mediate GLP-1R-induced body weight loss are currently being investigated. Here, we review mechanisms by which GLP-1R agonists reduce body weight.

### GLP-1R regulation of food intake

The reduction in body weight induced by GLP-1R agonists is largely attributed to a reduction in food intake. However, the specific sites that are involved in mediating GLP-1R induced hypophagia are not completely understood. Rats receiving vagotomy do not reduce food intake in response to peripherally injected GLP-1 indicating that vagal afferent nerves are needed to relay peripheral GLP-1 signals to higher brain regions to regulate feeding (Abbott et al., 2005). In contrast, another group demonstrated that peripheral administration of either liraglutide or Ex-4 induced a reduction in food intake in both control rats as well as rats that have had subdiaphragmatic vagal deafferentation (Kanoski et al., 2011). However, higher doses of liraglutide and Ex-4 were necessary in rats with subdiaphragmatic vagal deafferentation suggesting that vagal afferent neurons contribute to the suppression of food intake induced by peripherally administered GLP-1R mimetics but other brain regions are also involved in mediating this action (Kanoski et al., 2011). Another study used a genetic loss of function mouse model to delete the GLP-1R in vagal afferent/effect nerves (*Phox2b-Cre Glp1r^flox/flox^*) (Sisley et al., 2014). This study showed that chronic peripheral administration of liraglutide to *Phox2b-Cre Glp1r^flox/flox^* mice fed a HFD reduced food intake, body weight, and adiposity suggesting that the PNS is not required for liraglutide to mediate its effects on energy metabolism. Together these data indicate that although vagal afferent neurons may contribute to hypophagia induced by peripheral administration of GLP-1R analogs, these neurons are not necessary for the hypophagic effect.

It is evident that CNS GLP-1R signaling is essential for mediating the pharmacological action of GLP-1R agonists on energy metabolism as genetic deletion of CNS GLP-1R signaling in mice ablates the action of peripherally administered liraglutide on food intake and body weight reduction (Sisley et al., 2014). However, the specific brain regions that mediate these actions are not fully understood. The hindbrain has been implicated in mediating GLP-1R effects on food intake. Rats receiving brainstem-hypothalamic transection do not have a reduction in food intake in response to peripherally administered GLP-1 which suggests that the brainstem plays a critical role in communicating peripheral satiety signals to higher brain centers (Abbott et al., 2005). Furthermore, ICV injection of Ex-4 into the 4th ventricle of rats causes a suppression of food intake (Hayes et al., 2011). The specific brain regions in the hindbrain that mediate GLP-1R mediated suppression of food intake are currently being examined. The lateral parabrachial nucleus may be a critical site of action as pharmacological activation of GLP-Rs in the lateral parabrachial nucleus (LPBN) inhibits feeding whereas antagonism of LPBN GLP-1Rs induces hyperphagia (Alhadeff et al., 2014).

The hypothalamus has also been implicated as a major center for mediating the pharmacological effects of GLP-1R action on energy metabolism. More specifically, the ARC nucleus of the hypothalamus has been highlighted as one of the main sites mediating this action as chemical lesion of the ARC in rats ablates the anorectic action of GLP-1 (Tang-Christensen et al., 1998). Peripherally administered liraglutide has recently been shown to gain access into certain areas of the brain with the majority entering the ARC and median eminence with smaller amounts in the PVN (Secher et al., 2014). Animals that lack the GLP-1R do not show evidence of liraglutide in the ARC suggesting that peripherally administered liraglutide requires the GLP-1R to enter into the CNS (Secher et al., 2014). The specific neuronal populations in the ARC that mediate the anorectic action of GLP-1R signaling are currently being investigated. Much evidence suggests that activation of anorexogenic proopiomelanocortin (POMC) neurons and simultaneous inhibition of orexigenic neuropeptide Y/Agouti-related peptide (NPY/AgRP) neurons in the ARC appears to be one of the major mechanisms for GLP-1R mediated inhibition of food intake. In support of this, ICV administration of GLP-1 in rats has been shown to attenuate the fasting-induced rise in NPY/AgRP expression and decreases fasting-induced inhibition of POMC/CART expression in the ARC (Seo et al., 2008). Similarly, ICV injection of Ex-4 increased c-fos expression in alpha-melanocyte stimulating hormone (alpha-MSH)-immunoreactive neurons in the ARC of mice (Dalvi et al., 2012). In line with these data, the GLP-1R has been found to be co-expressed in ARC POMC neurons in both rats (Sandoval et al., 2008) and mice (Ronnekleiv et al., 2014) suggesting that GLP-1 can act directly on these neurons. Electrophysiological recordings support this notion and have demonstrated that GLP-1 directly stimulates POMC/CART cells, whereas GLP-1 can also inhibit orexigenic NPY/AgRP cells in the ARC through an indirect mechanism (Secher et al., 2014). Consistent with these findings, it has been demonstrated that the GLP-1R agonist Ex-4 (Ronnekleiv et al., 2014) excites ARC POMC neurons in mice. Taken together, the data in the literature indicate that GLP-1R signaling in ARC may be a major contributor to the homeostatic effects of GLP-1 analogs on feeding, whereas other brain regions such as the brainstem may play a minor role.

### GLP-1R mediated effects on visceral illness

Much debate exists around whether the suppression of feeding induced by GLP-1 and GLP-1 mimetics is solely a homeostatic effect. GLP-1 administration induces visceral illness in both rodents (Seeley et al., 2000) and humans (Madsbad et al., 2008). It is clear that the CNS is necessary for GLP-1R mediated induction of visceral illness as mice with a CNS deletion of the GLP-1R develop conditioned taste aversion (CTA) to lithium chloride but not to liraglutide (Sisley et al., 2014). The exact brain region mediating this action is still under investigation. Injection of GLP-1 directly into the central nucleus of the amygdala (CeA) of rats results in CTA (Kinzig et al., 2002) whereas injection into the PVN reduces food intake without eliciting CTA (McMahon and Wellman, 1998). However, other studies in rats show that injection of Ex-4 into the medial subnucleus of the NTS produced CTA whereas injection into the CeA did not induce CTA (Kanoski et al., 2012). Whether the discrepancy in results of these studies is due to the use of native GLP-1 vs. Ex-4 is unclear. Further studies are necessary to understand the mechanisms involved in GLP-1R induction of visceral illness.

### GLP-1R regulation of food reward

In addition to being expressed in hypothalamic areas that regulate homeostatic feeding, the GLP-1R is expressed in areas that mediate food reward in both rodents (Merchenthaler et al., 1999) and non-human primates (Heppner et al., 2015). Consistent with the neuroanatomical location, functional studies support a role for GLP-1R signaling in regulating food reward. Direct injection of Ex-4 into the nucleus accumbens (NAc) or ventral tegmental area (VTA) reduces food reward without causing visceral illness (Dossat et al., 2011; Dickson et al., 2012). Peripheral administration of liraglutide decreased preference for highly palatable foods in rats suggesting that GLP-1R mimetics may have therapeutic potential to decrease food reward (Raun et al., 2007; Hansen et al., 2012). To further support a role for GLP-1R signaling in regulating food reward, recent fMRI studies in obese and obese T2DM patients have demonstrated that intravenous infusion of exenatide decreases the activation of brain regions involved in mediating food reward (van Bloemendaal et al., 2014).

### GLP-1R action on brown adipose tissue (BAT) thermogenesis

In addition to causing changes in feeding, central GLP-1R activation may regulate body weight by increasing brown adipose tissue (BAT) thermogenesis (reviewed in Lockie et al., 2013). Chronic ICV administration of oxyntomodulin, a GLP-1R agonist, in mice decreased body weight despite having no significant effects on food intake (Lockie et al., 2012). Interestingly, BAT temperature was increased in these animals, which may have contributed to the weight loss. The effect of oxyntomodulin on body weight and BAT temperature is not evident in mice that lack the GLP-1R highlighting a GLP-1R dependent action. To determine the site of GLP-1R mediated action on BAT thermogenesis, another group performed intranuclear injections of liraglutide and determined that the ventromedial nucleus of the hypothalamus (VMH) is the specific brain region that mediates GLP-1R-induced BAT thermogenesis (Beiroa et al., 2014). Furthermore, this effect involves hypothalamic AMPK inhibition as pharmacological and genetic activation of AMPK ablates the effect of liraglutide on BAT thermogenesis. The extent to which GLP-1R activation of BAT contributes to weight loss in humans is still a matter of debate. Many studies indicate that energy expenditure in humans treated chronically with GLP-1R agonists experience no change (Harder et al., 2004; Bradley et al., 2012) or a decrease in energy expenditure (van Can et al., 2014). However, another study reported that a group of T2DM patients treated with metformin plus liraglutide or metformin plus Ex-4 for 1 year experience an increase in resting energy expenditure compared to placebo treated controls when energy expenditure was adjusted for fat free mass (Beiroa et al., 2014). However, from the data provided in that study, it is not possible to determine the contribution of BAT thermogenesis to the reported increase in energy expenditure. Thus, although the increase in CNS-GLP-1R signaling is sufficient to activate BAT thermogenesis, whether this regulation is necessary for the contribution of BAT thermogenesis to the control of energy balance, or to the body weight lowering effects of GLP-1 based therapies still requires further investigation.

### GLP-1R action on neuroprotection

GLP-1R analogs have neuroprotective and anti-inflammatory properties, which may be contributing the beneficial effects on energy metabolism (reviewed in Holscher, 2014). Studies in rodents have demonstrated that GLP-1R agonists reduce hippocampal cell death by reducing the development of amyloid-β plaques (McIntyre et al., 2013). This neuroprotective action in hippocampal cells may be one of the mechanisms whereby GLP-1R signaling enhances learning and memory in rodent studies (Oka et al., 1999; During et al., 2003; Abbas et al., 2009; McClean et al., 2010, 2011; Han et al., 2013). In addition to acting on hippocampal cells, chronic treatment with GLP-1 reduced degeneration of dopaminergic neurons in the substantia nigra and led to an improvement in motor function (Bertilsson et al., 2008; Harkavyi et al., 2008; Li et al., 2009). Although many preclinical studies suggest that GLP-1R agonism can act directly on neurons to prevent cell death, this neuroprotective property may be secondary to reduced glucotoxicity and inflammation in the brain that can result from weight loss and improvements in glycemic control (Berkseth et al., 2014).

## Effectiveness of GLP-1R agonists on weight loss in humans

Despite not have a complete understanding of the underlying molecular mechanisms, GLP-1R activation clearly has beneficial effects on both energy and glucose metabolism across a number of species. Moreover, patients with T2DM have impaired incretin action, which highlights GLP-1 as a useful pharmacotherapy for restoring glucose control (reviewed in Madsbad, 2014). Unfortunately, GLP-1 is rapidly degraded by the enzyme dipeptidyl peptidase-4 (DPP-4), which limits the therapeutic efficacy of native GLP-1 (Deacon et al., 1995). Therefore, synthetic GLP-1 analogs with resistance to degradation by DPP-4 were developed. There are currently two classes of GLP-1R analogs that are currently prescribed for the treatment of T2DM in humans: (1) GLP-1R agonists and (2) DPP-4 inhibitors (Drucker and Nauck, 2006).

### GLP-1R agonists

The GLP-1R agonists mediate their action by acting directly on the GLP-1R. Exenatide (Byetta™) was the first GLP-1R agonist to be prescribed for the treatment of T2DM and is a synthetic analog of the GLP-1R agonist, Ex-4. Ex-4 was originally isolated from the venom of *Heloderma suspectum* and shares 53% sequence homology with native GLP-1 (Eng et al., 1992). Exenatide has a relatively short half-life, (60–90 min in humans, Kolterman et al., 2005) and is recommended for twice-daily administration. Liraglutide (Victoza™) has 97% sequence homology with native GLP-1 and contains an Arg34Lys substitution as well as a glutamic acid and palmitic acid attached to Lys26. The addition of the fatty acid side chain to the peptide molecule allows for liraglutide to bind to albumin, which increases the time that the drug remains in circulation (Knudsen et al., 2000). The half-life for liraglutide is about 10–14 h in humans (Agerso et al., 2002) and is recommended for once-daily administration. Both exenatide (DeFronzo et al., 2005; Heine et al., 2005; Moretto et al., 2008; Norris et al., 2009) and liraglutide (Astrup et al., 2009; Garber et al., 2009; Niswender et al., 2013; Lean et al., 2014) significantly improve glycemic control and cause a significant body weight loss in T2DM patients. Gastrointestinal side effects including nausea and vomiting have been reported with both exenatide and liraglutide treatment although these side effects are often transient and occur mainly during the first few weeks of treatment in the dose escalation phase. A 26-week clinical study comparing the effectiveness of liraglutide (1.8 mg once-daily) with exenatide (10 μg twice-daily) indicates that patients treated with liraglutide had significantly greater reduction in HbA1c levels as well as significantly greater reduction in fasting plasma glucose levels as compared to patients treated with exenatide (Buse et al., 2009). However, both groups experienced similar levels of weight loss (−3.24 vs. −2.87 kg, liraglutide vs. exenatide).

A long-acting exenatide compound [exenatide extended release (ER; Bydureon™)] was synthesized by encapsulating exenatide into microspheres of medical-grade poly-(*D*, *L*-lactide-co-glycolide) which enables the drug to be released over an extended period of time (DeYoung et al., 2011). ER is recommended for once-weekly injection, which has major advantages in dealing patients who routinely forget to take their medication (Scott, 2012). The effects of ER once-weekly have been compared to exenatide twice-daily and overall ER was more effective at improving glycemic control and caused less gastrointestinal side-effects although both compounds caused a similar degree of weight loss (Drucker et al., 2008; Blevins et al., 2011).

The efficacy of once-weekly ER (2 mg) was compared to that of once-daily liraglutide (1.8 mg) in a 26-week trial in T2DM patients (Buse et al., 2013). Both treatments significantly improved glucose homeostasis and caused body weight reduction although liraglutide treated patients experienced a greater reduction in HbA1c levels, fasting serum glucose as well as a greater reduction in body weight as compared to ER. Adverse side effects including nausea, diarrhea and vomiting occurred more frequently in the liraglutide treated patients.

### DPP-4 inhibitors

The DPP-4 inhibitors mediate their action by inhibiting the enzyme DPP-4, which prevents the breakdown of GLP-1 and thereby increases endogenous GLP-1 levels. Sitagliptin (Aschner et al., 2006), saxagliptin (Rosenstock et al., 2009), and vildagliptin (Keating, 2010) all improve glucose metabolism in diabetic patients. One of the advantages of DPP-4 inhibitors is that patients rarely report nausea during treatment (Madsbad et al., 2008; Williams-Herman et al., 2010). However, unlike GLP-1R agonists, DPP-4 inhibitors do not cause significant reductions in body weight (Meneghini et al., 2011; Aroda et al., 2012). Taken together, GLP-1R agonists provide a superior advantage of reducing body weight in T2DM patients. However, T2DM patients with a lower tolerability for GLP-1R agonists may prefer treatment with DPP-4 inhibitors for the management of hyperglycemia.

## Novel GLP-1R co-therapies

The GLP-1R agonists exenatide and liraglutide have promising effects on both glucose and energy homeostasis in T2DM patients. However, most effects on body weight are modest and tend to range between 2 to 4% body weight reduction (reviewed in Davidson, 2013). For obese individuals, there is a need for a more powerful weight loss option. By combining GLP-1R agonism with other methods of weight loss, overall weight reduction can be enhanced. Here, we review therapies that combine GLP-1R pharmacotherapeutics with other weight loss mechanisms, which result in superior effects on body weight reduction and glucose metabolism.

### GLP-1R agonism in combination with the adjustable gastric band

Bariatric surgery is by far the most effective therapy for weight loss reduction and improvements in glucose metabolism (Pories, 2008). The two most effective bariatric procedures, Roux-en-Y gastric bypass (RYGB) and vertical sleeve gastrectomy (VSG) both result in enhanced meal-stimulated GLP-1 release which may be contributing to improvements in glucose metabolism and weight loss induced by these procedures. However, these types of bariatric surgery are very invasive and involve an irreversible manipulation of the gastrointestinal tract (reviewed in Stefater et al., 2012). A less invasive bariatric surgery is the adjustable gastric band (AGB), which involves placing an inflatable silicon band around the stomach near the esophageal junction. To modulate the degree of restriction, the band is inflated by infusing saline into a subcutaneously implanted port. Unfortunately, the AGB is far less effective on weight loss as compared to RYGB and VSG (Buchwald et al., 2009), which may be partially due to the fact that AGB does not enhance circulating GLP-1 levels (le Roux et al., 2006). A study in rats demonstrated that AGB can act synergistically with the GLP-1R agonist, Ex-4, to promote a more substantial weight loss as compared to Ex-4 treatment or AGB alone (Habegger et al., 2013). The advantage of this approach is that it combines two minimally invasive and reversible weight loss strategies, which in the future may provide patients with an alternative approach to weight loss.

### Single molecule multi-agonists

A number of metabolic hormones can achieve favorable effects on body weight loss but cannot be used as a weight loss therapeutic because of negative side effects. A new generation of pharmacotherapies have been developed which combine GLP-1R agonism with other metabolic hormones into a single molecule. This new line of pharmaceuticals maximizes the beneficial effects on energy and glucose metabolism while minimizing unwanted side-effects (Tschop and DiMarchi, 2012).

Glucagon (Gcg) has many beneficial effects on energy homeostasis including the inhibition of food intake, as well as the ability to increase energy expenditure and activate BAT which all lead to a reduction in body weight (reviewed in Habegger et al., 2010; Heppner et al., 2010). The fact that Gcg causes an elevation in blood glucose levels in combination with its limited solubility at a physiological pH has discouraged the use of glucagon as a therapeutic for weight loss. Single molecules with GLP-1 and Gcg co-agonism were created to take advantage of the beneficial effects of both GLP-1 and Gcg on energy metabolism while limiting Gcg induction of hyperglycemia (Day et al., 2009; Pocai et al., 2009). Diet-induced obese (DIO) mice treated chronically with the GLP-1/Gcg coagonists lost significant body weight and fat mass and interestingly, also had improved glucose metabolism. However, *Glp1r−/−* mice treated with the GLP-1/Gcg coagonist experienced a reduction in body weight and fat mass but did not have improvements in glucose tolerance indicating that GLP-1R agonism is required to counteract the hyperglycemic effects of GcgR agonism (Day et al., 2009). The beneficial effects of single molecule GLP-1/Gcg coagonism were also demonstrated in ob/ob mice, which had enhanced GSIS and improved glucose tolerance upon acute treatment with a GLP-1/Gcg coagonist (Gault et al., 2013). To determine the site of action in the brain that GLP-1 and Gcg coagonism may be occurring, one group examined c-fos induction upon co-injection of GLP-1 and Gcg (Parker et al., 2013). Doses of GLP-1 and Gcg that when administered alone did not affect food intake were given as a co-injection which resulted in a significant reduction in feeding and c-fos induction in both the area postrema and CeA. Whether the single molecule GLP-1/Gcg coagnonist has a similar CNS pattern of activation requires further investigation. Similar beneficial effects of GLP-1/Gcg coagonism on energy and glucose metabolism have been demonstrated in humans. Although not used in a single molecule, healthy human volunteers showed increased energy expenditure upon coinfusion of GLP-1 and Gcg, which was not apparent with GLP-1 infusion alone (Tan et al., 2013). Additionally, GLP-1/Gcg coinfusion limited the hyperglycemic action of Gcg (Tan et al., 2013). These data give a promising outlook that the enhanced effectiveness of GLP-1/Gcg coagnoism demonstrated in rodents will translate to enhanced weight loss in humans.

Glucose-dependent insulinotropic polypeptide (GIP) is an incretin hormone released from the K-cells of the small intestines and acts on the pancreas to potentiate GSIS (Miyawaki et al., 1999). Unlike, GLP-1 and Gcg, GIP is not derived from preproglucagon and does not cause a reduction in body weight (Baggio and Drucker, 2007). Interestingly, by combining GLP-1 and GIP agonism into a single molecule, the GIP/GLP-1 coagonist has enhanced therapeutic properties to improve glucose tolerance and induce weight loss, which was demonstrated in rodents, non-human primates and humans (Finan et al., 2013). Finally, a triagonist was synthesized to include GLP-1, Gcg, and GIP agonism (Finan et al., 2015). As compared to mono or coagonists, the triagonist had enhanced effectiveness to decrease body weight and fat mass in rodents. This effect was a result of a combination of a reduction in food intake and a shift in metabolic fuel preference to favor fat utilization.

An alternative approach aiming to maximize the efficacy of GLP-1 involves the delivery of nuclear receptor agonists to specific tissues characterized by the expression of GLP-1R. The superior efficacy of a single molecule combining GLP-1 and estrogen receptor activity improving metabolic control supports the feasibility of this approach. Estrogen is a steroid hormone with beneficial effects in the control of energy balance (Brown and Clegg, 2010; Mauvais-Jarvis et al., 2013; Frank et al., 2014), however, these properties cannot be taken full advantage of because estrogen also acts as a carcinogen (Huang et al., 2014). By stably linking estrogen to GLP-1 in a single molecule, this coagonist can take advantage of estrogen's effects on weight loss while avoiding the negative side effects on other tissues (Finan et al., 2012). DIO ovariectomized female mice treated chronically with a stable estrogen/GLP-1 coagonist had significantly greater body weight and fat mass loss as compared to GLP-1R monagonists. Neither proliferation of the uterine lining nor tumor growth were detected in animals treated with the stable co-agonist indicating that the molecule was not having negative off target effects on peripheral tissue. The estrogen/GLP-1 coagonist loses its potency of action when administered to animals with a CNS specific deletion of the GLP-1R indicating that the GLP-1R is essential for full effectiveness. The superior beneficial effects of GLP-1/estrogen co-agonism were also demonstrated in a mouse model prone to the development of diabetes, the New Zealand obese (NZO) mouse. In contrast to GLP-1 mono-therapy, GLP-1/estrogen co-agonism prevented hyperphagia and beta-cell failure in NZO mice (Schwenk et al., 2014). Taken together, the addition of other metabolic hormones to GLP-1R agonism results in superior effects on glucose and energy homeostasis.

## Conclusion

**Figure 1:**
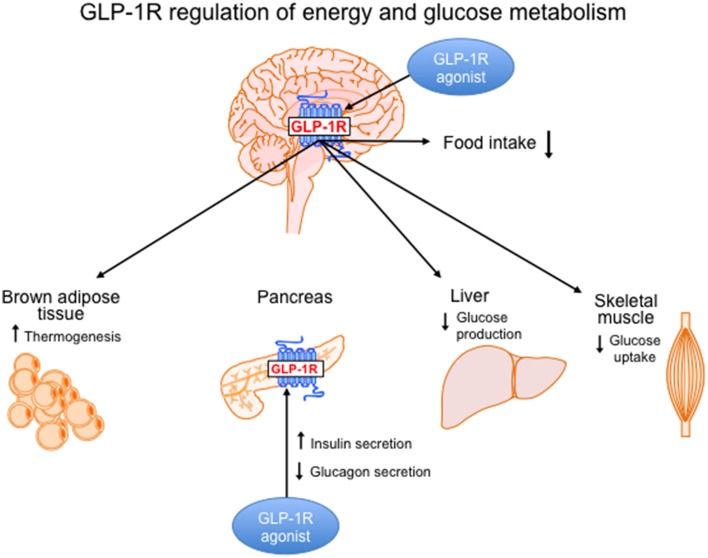
**GLP-1R signaling in the brain and periphery regulates energy and glucose metabolism**. Body weight loss induced by GLP-1R agonism is controlled by CNS-mediated mechanisms and is mainly a result of a reduction in food intake. However, GLP-1R action in the brain activates brown adipose tissue thermogenesis, which may also contribute to weight loss. In the brain, GLP-1R signaling regulates glucose homeostasis by decreasing hepatic glucose production and decreasing glucose uptake in muscle. In the periphery, GLP-1R agonists act directly on the pancreas to increase insulin secretion and reduce glucagon secretion.

In addition to the well-established role of GLP-1 to improve glucose homeostasis, GLP-1R agonism has beneficial effects on body weight reduction. The tissue-specific effects of GLP-1R signaling on energy and glucose and energy homeostasis are illustrated in Figure 1 and are divided into CNS vs. peripheral GLP-1R-mediated action. The fact that GLP-1R agonists have beneficial effects on both energy and glucose metabolism places this class of pharmacotherapies at a superior level compared to other drugs used to treat T2DM that cause weight gain. Although physiological levels of GLP-1 can improve glycemic control, pharmacological levels of GLP-1 must be reached to promote weight loss. This is demonstrated by comparing the effects of DPP-4 inhibitors vs. GLP-1R agonists in T2DM patients. Both classes of drugs improve glycemic control in T2DM patients but only the GLP-1R agonists given at pharmacological doses will cause weight loss. However, the weight loss induced by GLP-1R mono-therapies in obese T2DM patients is modest indicating a need for more robust therapeutic options. Pre-clinical data from studies that combine GLP-1R agonism with other weight loss strategies such as AGB or other metabolic hormones have highlighted more potent options for weight loss. Clinical testing of these combination therapies will be necessary to determine whether the effects translate to superior weight loss in obese T2DM patients.

### Conflict of interest statement

The authors declare that the research was conducted in the absence of any commercial or financial relationships that could be construed as a potential conflict of interest.
